# Forest-Going as a Risk Factor for Confirmed Malaria in Champasak Province, Lao PDR: A Case-Control Study

**DOI:** 10.3390/ijerph21121624

**Published:** 2024-12-04

**Authors:** Sarah Gallalee, Emily Dantzer, Francois Rerolle, Keobouphaphone Chindavongsa, Khampheng Phongluxa, Wattana Lasichanh, Jennifer L. Smith, Roly Gosling, Andrew Lover, Bouasy Hongvanthong, Adam Bennett

**Affiliations:** 1 Malaria Elimination Initiative, Institute for Global Health Sciences, University of California San Francisco, San Francisco, CA 94158, USA; 2 Department of Epidemiology and Biostatistics, University of California San Francisco, San Francisco, CA 94158, USA; 3 Center for Malariology, Parasitology and Entomology, Ministry of Health, Vientiane 01000, Laos; 4 Lao Tropical and Public Health Institute, Ministry of Health, Vientiane 01000, Laos; 5 Department of Disease Control, London School of Hygiene and Tropical Medicine, London WC1E 7HT, UK; 6 Department of Biostatistics and Epidemiology, School of Public Health and Health Sciences, University of Massachusetts-Amherst, Amherst, MA01003, USA

**Keywords:** forest-goers, high-risk populations, Lao PDR, malaria, *Plasmodium falciparum*, *Plasmodium vivax*, surveillance

## Abstract

Lao People’s Democratic Republic (Lao PDR) has made significant progress in reducing malaria in recent years. In the Greater Mekong Subregion, forest-going is often a risk factor contributing to continuing malaria transmission. This study assessed forest-going and other potential risk factors for malaria cases in Champasak Province, Lao PDR. Routine passive surveillance data from August 2017 to December 2018 were extracted from health facilities in three districts for a case-control study; at the time of presentation, all fever cases were asked to report any recent forest travel. Multivariable logistic regression was used to assess the relationship between forest-going and malaria infection while controlling for other covariates. Of 2933 fever cases with data available on forest-sleeping and malaria diagnosis from 25 health facilities, 244 (8%) tested positive (cases), and 2689 (92%) tested negative (controls). Compared with spending 0–2 nights in the forest, spending 3–7 nights in the forest was associated with 9.7 times the odds of having a malaria infection (95% CI: 4.67–20.31, *p* < 0.001) when adjusting for gender, occupation, and season. Forest-going, especially longer trips, is associated with increased risk for confirmed symptomatic malaria in southern Lao PDR, and appropriate and targeted intervention efforts are needed to protect this high-risk population.

## 1. Introduction

The Greater Mekong Subregion (GMS), including Cambodia, Lao PDR (People’s Democratic Republic), Myanmar, Thailand, Vietnam, and Yunnan Province of China, has become a focal point of global malaria work in recent years because drug resistance to artemisinin-based combination therapies (ACTs) has spread, threatening global control efforts and making malaria elimination more urgent [1]. With this increased focus on elimination, countries in the GMS reduced the regional reported number of malaria cases by 74% between 2012–2018 [2]. Lao PDR has made significant progress in reducing the malaria burden in recent years; confirmed cases fell 81% from 46,202 in 2012 to 8913 in 2018 [3]. In the context of increasing resistance to ACTs in the subregion, the country aims to eliminate all species of malaria by 2030.

Malaria is a vector-borne disease transmitted by female *Anopheles* mosquitoes and caused by multiple species of *Plasmodium* parasites. The clinical manifestations of uncomplicated malaria are typically fever, chills, and sweating and sometimes include vomiting, headache, fatigue, body aches, and nausea; if the disease progresses to severe malaria, complications can include seizures, shock, severe anemia, cardiovascular collapse, and respiratory distress [4]. Common diagnostic methods include microscopy, rapid diagnostic testing (RDT), loop-mediated isothermal amplification (LAMP), and polymerase chain reaction [5]. The World Health Organization’s technical strategy for malaria includes three pillars: ensuring universal access to prevention, diagnosis, and treatment for malaria, increasing global efforts toward the elimination of malaria, and ensuring that malaria surveillance is a core intervention [6].

In the malaria elimination setting of the GMS, there is substantial evidence that the population at greatest risk for malaria is adult males who travel and work in forested areas [7,8,9]. These individuals are often exposed to the predominantly outdoor-biting, forest-adapted malaria vectors in the region through work such as logging, agriculture, forest foraging, and industrial work, and transmission in these populations risks sustaining country and region-wide malaria transmission [7]. Traditional malaria control interventions such as long-lasting insecticide-treated nets (LLINs) and indoor residual spraying (IRS) do not protect individuals who travel and sleep outside at night, and long-lasting insecticide-treated hammocks (LLIHs) are often not widely accessible or are not appropriate when individuals are moving or working at night [10,11]. Additionally, some of these workers are highly mobile or migrant, which often leads to decreased access to healthcare [12,13]. It has been a continuing challenge in the region to understand the best interventions to protect these populations from malaria transmission. Determining who is at risk, characterizing the populations at risk in more detail, and assessing which interventions are most effective for them are priorities for elimination [14].

In Lao PDR, forest-based activities are a major part of the economy, and between 2009 and 2014 (during a period of heavy forest activity and a major malaria outbreak), the proportion of reported malaria cases among males aged 5 years and older increased from 46% to 86%, which may have resulted from an increased proportion of infections due to occupational exposures [8]. Despite the growing consensus in the GMS that adult forest-going men are at the highest risk for malaria, it is still essential to delineate specific sub-regional patterns and risk factors. A better understanding of the association between specific types of travel and sleeping in the forest and malaria risk, as compared to village-based risk, is needed to choose appropriate targeted interventions to eliminate malaria transmission in Lao PDR and the region.

This study assessed whether the length of time spent overnight in the forest was a risk factor for confirmed malaria cases compared to malaria-negative fever controls diagnosed through the health system in a higher-burden province of southern Lao PDR. Specifically, routinely collected malaria surveillance data were supplemented to include potential risk factors related to forest-going to determine if there was an association between staying overnight in the forest and malaria risk in Champasak Province, Lao PDR. Much of the contents of this paper have been drawn from a dissertation chapter from 2023; this work has not been published in any other journal.

## 2. Materials and Methods

### 2.1. Study Area

**Figure 1:**
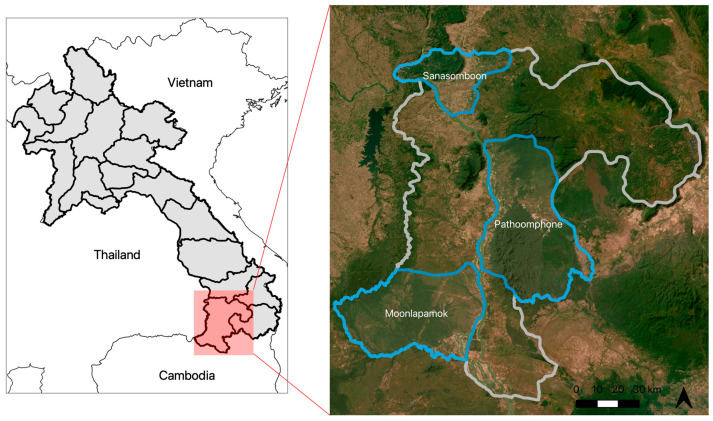
Three study districts in Champasak Province, Lao PDR.

Champasak Province is located in southern Lao PDR and is bordered by Thailand, Cambodia, and three other provinces in Lao PDR (Figure 1); as of 2018, 95% of the country’s malaria burden occurred in the five southernmost provinces in Lao PDR [15]. Studies in Cambodia have found evidence of artemisinin resistance, and border provinces such as Champasak have been of high priority for malaria elimination [1]. Monsoon season spans from June to October [16,17]. Champasak is a heavily forested province that is moderately mountainous with an economy mainly reliant on agricultural and forest-based work [16]. As of the 2015 census, the population of Champasak was 694,023, and the population density was 45 people per square kilometer [18]. In Lao PDR, three species of *Anopheles* are considered the major vectors: *Anopheles dirus*, *An. maculatus*, and *An. minimus* [19]. The study took place in three districts: Mounlapamook, Panthampone, and Sanamsaboun, which were part of a larger study area of a randomized controlled trial (RCT) aimed at assessing the effectiveness of active case detection in southern Lao PDR [20]. In 2015, the respective population and annual parasite indices (API) of Mounlapamook, Panthampone, and Sanamsaboun were 38,800; 61,252; 69,338 and 66.8; 83.0; and 26.8 per 1000 population.

### 2.2. Study Design

During the study period of the RCT on active case detection [20], health facility workers were trained to collect augmented routine malaria surveillance data in order to capture additional variables related to forest behavior. In Lao PDR, guidelines indicated that any febrile patient should be considered a suspected case and tested for malaria. The study population was drawn from suspected patients tested for malaria at 25 selected health facilities that were part of the study in three districts; all febrile patients with suspected malaria who received a malaria test at 25 health facilities were included in the study. Patients who tested positive for malaria using a rapid diagnostic test (RDT) or microscopy were considered a case; patients who tested negative for malaria using a RDT or microscopy were considered a control. The RDTs used were the same that were used nationally by the Center for Malariology, Parasitology, and Entomology: CareStart Ag Pf/Pv (SD Bioline, Cat #05FK80, Seoul, Republic of Korea) [5,21].

### 2.3. Data Collection

Outpatient register data from the paper-based (at the health facility level) passive malaria surveillance system were collected from 25 health centers for the period between August 2017 and December 2018 (17 months). Participants were those who visited health facilities in three districts of Champasak Province and were tested for malaria with a diagnostic test (RDT or microscopy). The registries included data on the date of diagnosis, species-specific test results, demographic information (age, gender, occupation), village of residence, and the added questions on forest-going behavior (number of days slept in the forest in the past 30 days, suspected place infected with malaria, and travel destinations in the past 30 days).

### 2.4. Data Processing

Data cleaning and analysis were conducted using STATA 16.1 and R 4.1.2. Participants were included if they had a malaria test result and available data on the main variable of sleeping in the forest. One percent of participants were missing demographic information and were removed from the analysis. One mixed-infection malaria case was included in the *Plasmodium falciparum* species category. Categories within variables that were conceptually similar were recombined to avoid cells smaller than five, including age groups (1–15 and >15) and occupation. (e.g., age group and occupation). Occupation was grouped into three groups: farmer, student/child, and other. Other categories included housewife, employee, monk, and retired. A categorization of occupation with a larger number of groups was not possible due to small cell sizes. An analysis comparing sleeping in the forest to sleeping no nights in the forest was not possible due to small cell sizes (only one malaria case slept no nights in the forest, so sleeping 0–2 nights was used as the reference). Seasons were defined as the dry season (November–May) and monsoon season (June–October) [17].

### 2.5. Data Analysis

Associations between risk factors and the outcome of malaria infection were explored using unconditional logistic regression with a clustering term at the health facility level. Model building was informed by a directed acyclic graph (DAG) conceptualizing relationships between forest exposure (sleeping in the forest), the outcome of malaria infection, and potential confounding variables [22]. We then assessed the bivariate relationship of the variables identified as potential confounders (age, gender, occupation) between the main exposure and the outcome. Crude odds ratios (ORs) with 95% confidence intervals were estimated in bivariate logistic regression; variables associated with the outcome (significant at *p*-value 0.05) or those that changed the main effect OR by more than 10% in the final model were included. The same analysis approach was used to assess the outcomes of *P. falciparum* and *P. vivax* separately. Sensitivity analyses were carried out to evaluate the potential impacts of the missing data on forest-sleeping behavior. Two separate models were compared, one where all patients with missing data on forest-sleeping were assigned to having slept 3–7 nights in the forest, and a second where they were assigned zero nights.

## 3. Results

Between August 2017 and December 2018, 6239 patients with a malaria test result and demographic information were studied using routine surveillance at 25 health facilities in the three study area districts. 3306 (24% of cases and 55% of controls) were missing a response for the variable number of nights spent sleeping in the forest in the past 30 days; these were dropped from the main analysis (Supplementary Table S1). A total of 2933 patients were included in the analysis: 244 (8%) were positive for malaria. Among malaria-positive cases, 44% were *P. falciparum,* 56% were *P. vivax*, and one case was a mixed infection. The distribution of potential risk factors for malaria among participants is presented in Table 1.

**Figure 2:**
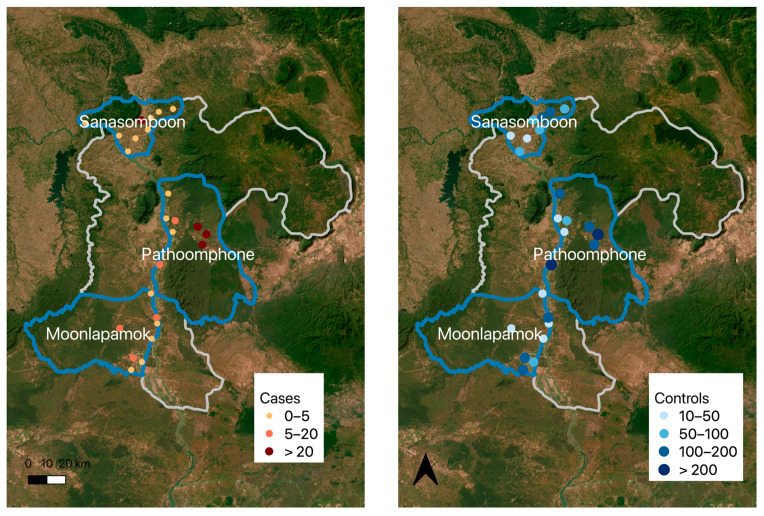
Cases and controls by health facility.

The majority of participants were over 15 years old (86%), male (82% of cases and 66% of controls), and reported their primary occupation as farmers (78%) (Table 1). The majority of cases were in the Panthampone district (Figure 2). In the Sanasomboun district, 31 out of 32 cases were identified as a farmer, and 100% of cases were over 15 years old. Five participants under 10 years old had malaria (one of them was under 5 years old). The occupation category of “other” included 49 participants who identified as housewives (all negative for malaria) and 77 who identified their jobs as employees (12% were positive). Among cases, 72% of women and 92% of men were over 15 years of age (80% and 87%, respectively, among the controls).

Only one person who was malaria positive (for *P. falciparum*) reported not sleeping in the forest during the last 30 days. Of patients with forest travel data available, 99.6% of positives and 71% of controls reported sleeping at least 1 night in the forest in the last 30 days.

**Figure 3:**
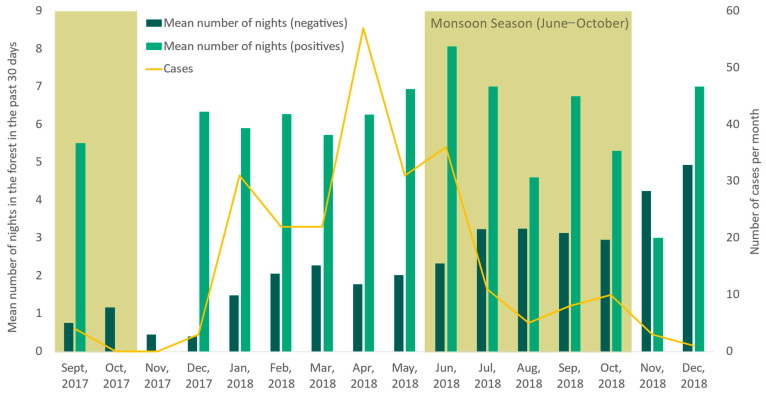
Total cases and average number of nights spent in the forest in the past 30 days among participants who were positive or negative for malaria.

The average length of trip was similar among the under 15 years old and over 15 years old age groups and among men and women in both cases (average 6.4 nights in the forest, standard deviation: 4.7) and controls (average 2.3 nights in the forest, standard deviation: 2.6). As shown in Figure 3, the number of nights spent in the forest was similar on average during monsoon season (average: 3, SD: 3, min: 0, max: 30) compared to dry season (average 2.5, SD: 2.9, min: 0, max: 16; *p* < 0.005). The average number of nights spent in the forest did not differ significantly between cases of *P. falciparum* and *P. vivax* (Table 1); however, season did: 92% of *P. falciparum* cases presented during dry season compared to 53% of *P. vivax* cases.

Compared with sleeping 0–2 nights in the forest in the past 30 days, sleeping 3–7 nights in the forest led to 9.74 times the odds of having malaria (95% CI: 4.67–20.31, *p* < 0.001) when adjusting for gender, occupation, and season (Table 2). The odds of having malaria were increasingly higher for longer periods of sleeping in the forest; with 8–14 nights and over 14 nights associated with 38.7 (95% CI: 12.42–120.67, *p* < 0.001) and 43.67 (95% CI: 5.92–322.33, *p* < 0.001) times the odds of having malaria compared to 0–2 nights, respectively. The results from the sensitivity analyses suggested that these relationships remained unchanged despite estimating the results if those missing data on forest-sleeping had slept in the forest or had not slept in the forest (Supplementary Table S2). Additionally, in the adjusted model, men had 2.23 (95% CI: 1.21–4.08, *p* < 0.05) times the odds of having malaria than women. Patients also had 1.97 (95% CI: 1.04–3.70, *p* < 0.05) times the adjusted odds of getting malaria during the dry season (November–May) compared to during the monsoon season.

These trends remained similar when *P. falciparum* and *P. vivax* cases were analyzed separately in subgroup analyses. Those who spent 3–7 nights in the forest in the past 30 days had had 11.23 (95% CI: 4.49–28.06, *p* < 0.001) times the adjusted odds of *P. falciparum* malaria and 8.83 (95% CI: 3.78–20.60, *p* < 0.001) times the adjusted odds of *P. vivax* malaria than those who slept 0–2 nights in the forest (Table 2). Men had 1.74 (95% CI: 0.97–3.11, *p* < 0.062) times the adjusted odds of *P. falciparum* malaria and 2.74 (95% CI: 1.34–5.62, *p* < 0.01) times the adjusted odd of *P. vivax* malaria than women. While dry season was associated with higher odds of *P. falciparum* malaria than monsoon season (aOR: 8.8, 95% CI: 4.05–18.70, *p* < 0.001), for *P. vivax* there was no evidence of an association (Table 2).

## 4. Discussion

Sleeping in the forest, especially during longer trips, was associated with an increased risk for symptomatic facility-confirmed malaria infection in southern Lao PDR. Those who spent more than 2 weeks in the forest in the past 30 days had 44 times the adjusted odds of having malaria (95% CI: 5.92–322.33, *p* < 0.001) compared to those who spent 2 nights or less in the forest. Men had twice the adjusted odds of having malaria compared to women (95% CI: 1.21–4.08, *p* < 0.05). These results are consistent with a body of literature suggesting that working-age males who are exposed to outdoor-biting mosquitoes while taking extended trips to the forest are at high risk for malaria in the GMS [7,8,14,23,24]. Studies have found that forest-going behavior in Lao PDR includes logging, collecting timber or bamboo, hunting, foraging for food or plants, agricultural work such as rice farming, and military activities [16,17,25,26]. In this study, most participants identified their main occupation as farming (though the answer can be season-dependent); other studies have also reported that farmers are a group who often stay overnight in the forest [26,27]. In formative work in the study districts, we found that rice farmers often spend nights in huts close to the forest fringe, whereas individuals engaging in forest activities often slept or worked through the night in the forest [27]. In the limited data available on the reason for travel, participants reported going to the forest to cut wood or look for food or fruit. Additional research could assess the malaria risk associated with specific occupational groups that work in the forest.

Only one malaria case out of 244 did not spend any nights in the forest in the past 30 days. Of the 49 women who identified as housewives, none were positive for malaria, and of the 69 children under 5 years old, only 1 was positive, which could suggest minimal community transmission of malaria in villages of the study area. This finding is supported by the results of the main trial on active case detection from the randomized controlled trial over the same study period [20]. These results support the need to target appropriate interventions to forest workers and locations rather than village locations and understand the proportion of forest-goers based in villages near the forest [17]. In this study, trips to the forest were varied in duration and season; this is supported by literature that has noted the heterogeneity in patterns of travel among forest-goers [28,29]. Challenges remain in understanding which tools are most effective and where, when, and how to access these populations.

Another important finding of this study is the relevance of seasonality to malaria transmission in Lao PDR. Spending the night in the forest was associated with similar odds for both *P. falciparum* and *P. vivax*; however, dry season was associated with higher odds for *P. falciparum* than monsoon season, while odds of *P. vivax* malaria were not higher during one season than the other. Additionally, 92% of *P. falciparum* cases were captured during the dry season compared to 53% of *P. vivax* cases. These findings could be explained by the relapsing behavior of *P. vivax* infections arising from the dormant liver stages [30], making it more difficult to determine when malaria was acquired by the patient. The higher risk of *P. falciparum* malaria during the dry season could be related to the human behavior of forest-goers (e.g., they are likely able to go deeper into the forest when it is dry), though the relationships are difficult to untangle [17]. Studies on vector bionomics in Lao PDR have found that people in remote and rural areas are regularly exposed to malaria vectors outside throughout both seasons [19,31]. In this study, the number of nights spent in the forest was longer during the monsoon season than the dry season, and other studies have found that forest-goers in Lao PDR may be more active during the monsoon season, e.g., drawn out to work and spend the night in rice fields [17]. However, the intensity of monsoon season can also lead to flooded and impassible roads, which could indicate less access to testing at health facilities during this season and could explain longer trips when mobility to go back and forth for brief stints is limited [32]. In one study among military personnel in Champasak, respondents reported that using nets in the forest was particularly challenging during monsoon season [10].

A goal of this study was to determine if risk factors for malaria in Champasak could be assessed utilizing routine surveillance data that had been augmented with additional forest-going questions. As routine data are regularly collected, utilizing them for research does not add additional work burdens to local staff, though there is a need for monitoring and supervision of staff to avoid challenges such as missing data. We determined that this type of study can be effective if the research aim is to confirm or reassess an individual risk factor that can be asked simply. However, for a more detailed characterization of risk populations, some key additional questions could be included, such as details on occupation, behavior, preventative methods, home location, and travel destinations. Future research could develop a more detailed characterization of forest-going activities and delineate risk amongst forest-goers depending on the risk level of forest-based activity in order to define intervention points [17,28,33].

One limitation of this study is that test positivity rates are highly dependent on treatment-seeking behavior at public health facilities and the quality of diagnostic testing practices. As a metric for malaria transmission, this indicator may be biased; for example, areas farther away from the clinic likely have less attendance, and the true burden of malaria may be underestimated because not everyone visits a public facility for febrile illness. Additionally, if seeking treatment is associated with sleeping in the forest, this could lead to selection bias. Data on treatment-seeking behavior and quality of diagnostic testing were not available to include in the models. Another limitation of this study was that a large percentage of responses for the forest-going variable were missing, and more were missing for controls than cases. It is likely that health facility workers were more concerned with gathering data on forest-going for the cases than for the controls, which could lead to differential misclassification of the exposure. However, the results of the sensitivity analyses on missing forest-going data indicated that the relationships between forest-sleeping and malaria remained strong and significant. An additional limitation is that variables available for this analysis were mostly limited to those available in Lao PDR’s passive surveillance system; therefore, additional confounding variables may exist that were not available for analysis. It was also not possible to adjust for environmental covariates due to the limited geolocation data of the villages and the lack of specifics and details in the open-response travel destination data. In the formative work, it was a challenge for the researchers to determine how to pose the questions of travel destination; the forest-goers are often moving frequently and unfamiliar with local areas as viewed on a map [27].

There is ample evidence that forest-going behaviors have an impact on malaria transmission in the GMS; the findings of this study show the higher risk of malaria associated with longer trips to the forest in Lao PDR and reinforce the need for control measures that can be effective for preventing malaria among forest-going populations who spend extended periods of time in the forest. Studies in the region have explored alternative interventions for these populations, such as insecticide-treated hammocks, protective clothing, mosquito coils, and mosquito repellants; however, there are shortfalls in these interventions, such as lack of effectiveness and desirability (e.g., when workers are hot and sweaty and working through the night) [28,34,35,36]. Enhanced case finding may also be important to target this population, as individuals may be less able (or willing) to seek care at health facilities; this could include mobile or border health units or community volunteers that are placed at key access points such as plantations or forest industry sites [37]. Chemoprophylaxis with anti-malarial medication is also a potential option; recent studies indicate that chemoprophylaxis can be effective and acceptable for forest-goers in Southeast Asia [28,38,39,40]. This study found that trips were highly heterogeneous in season, purpose, and length; this finding supports calls for appropriate interventions to be targeted based on the spatio-temporal variability in size and behavior of the forest-going populations [17,28,29].

## 5. Conclusions

Characterizing and gleaning a better understanding of forest-going populations and the malaria risk associated with trips to the forest, as well as targeting these populations with appropriate interventions, is essential to malaria elimination in the GMS. The large size of the forest-going populations, the diversity in the duration, activity, and seasonality of forest-going trips, and the vast and often hard-to-reach areas pose large challenges for targeting these groups with effective interventions for malaria prevention and treatment. Studies utilizing routine passive surveillance data can be useful for confirming suspected risk factors for malaria, and often, adding a few highly relevant variables can be useful in further understanding and defining high-risk populations. More extensive questionnaires can also be administered during routine surveillance to characterize forest-goers in enough detail to define intervention points [31], and additional approaches are needed to incorporate populations that do not reach healthcare at public facilities. Forest-going, especially longer trips, is associated with increased risk for malaria in southern Lao PDR; targeted and novel control efforts are needed to protect populations that work and sleep in the forest.

## Figures and Tables

**Table 1:** Characteristics of patients who visited health centers in three districts of Champasak, Lao PDR during the study period and had data available on forest-going. Percentages are row percentages to depict test positivity prevalence by group. The one mixed-infection case was grouped in the *P. falciparum* category.

Variable	Overall	Malaria Negative	Malaria Positive		
			All Positive	*P. falciparum*	*P. vivax*
	*n* = 2933	*n* = 2689	*n* = 244	*n* = 108	*n* = 136
District					
Mounlapamoak	691	651 (94%)	40 (6%)	9 (1%)	30 (4%)
Pathoumphone	1474	1302 (88%)	172 (12%)	98 (7%)	74 (5%)
Sanasomboun	768	736 (96%)	32 (4%)	0 (0%)	32 (4%)
Age group					
1–15 years	425	396 (93%)	29 (7%)	7 (2%)	22 (5%)
>15 years	2508	2293 (91%)	215 (9%)	100 (4%)	114 (5%)
Gender					
Male	1984	1783 (90%)	201 (10%)	86 (4%)	115 (6%)
Female	949	906 (95%)	43 (5%)	21 (2%)	21 (2%)
Occupation					
Farmer	2294	2085 (91%)	209 (9%)	102 (4%)	106 (5%)
Student and/or child	511	485 (95%)	26 (5%)	4 (1%)	22 (4%)
Other	128	119 (93%)	9 (7%)	1 (1%)	8 (6%)
Nights in the forest					
0–2	1724	1695 (98%)	29 (2%)	12 (1%)	17 (1%)
3–7	1041	894 (86%)	147 (14%)	66 (6%)	81 (8%)
8–14	139	83 (60%)	56 (40%)	25 (18%)	30 (22%)
>14	29	17 (59%)	12 (41%)	4 (14%)	8 (28%)
Season					
Dry	1678	1508 (90%)	170 (10%)	98 (6%)	72 (4%)
Monsoon	1255	1181 (94%)	74 (6%)	9 (1%)	64 (5%)

**Table 2:** Unadjusted (OR) and adjusted (aOR) odds ratios for bivariate and multivariable logistic regression.

All Species				
Variable	OR (95% CI)	*p* Value	aOR (95% CI)	*p* Value
Age group				
1–15 years	Reference		—	—
>15 years	1.28 (0.81–2.01)	0.285	—	—
Gender				
Female	Reference		Reference	
Male	2.38 (1.36–4.15)	<0.005	2.23 (1.21–4.08)	<0.05
Occupation				
Farmer	Reference		Reference	
Student and/or child	0.53 (0.31–0.93)	<0.05	0.67 (0.34–1.31)	0.244
Other	0.75 (0.13–4.31)	0.75	0.73 (0.14–3.64)	0.705
Nights in the forest				
0–2	Reference		Reference	
3–7	9.61 (4.68–19.75)	<0.001	9.74 (4.67–20.31)	<0.001
8–14	39.43 (13.09–118.81)	<0.001	38.71 (12.42–120.67)	<0.001
>14	41.26 (6.25–272.42)	<0.001	43.67 (5.92–322.33)	<0.001
Season				
Monsoon	Reference		Reference	
Dry	1.80 (0.88–3.66)	0.105	1.97 (1.04–3.70)	<0.05
***P. falciparum***				
**Variable**	**OR (95% CI)**	***p* value**	**aOR (95% CI)**	***p* value**
Age group				
1–15 years	Reference		—	—
>15 years	2.49 (0.88–7.03)	0.085	—	—
Gender				
Female	Reference		Reference	
Male	1.98 (1.13–3.49)	<0.05	1.74 (0.97–3.11)	0.062
Occupation				
Farmer	Reference		Reference	
Student and/or child	0.17 (0.07–0.39)	<0.001	0.21 (0.08–0.53)	<0.005
Other	0.17 (0.03–0.88)	<0.05	0.18 (0.02–1.31)	0.091
Nights in the forest				
0–2	Reference		Reference	
3–7	10.43 (4.24–25.68)	<0.001	11.23 (4.49–28.06)	<0.001
8–14	44.25 (12.62–155.13)	<0.001	41.24 (10.43–163.03)	<0.001
>14	33.24 (4.32–255.70)	<0.005	31.64 (4.13–242.74)	<0.005
Season				
Monsoon	Reference		Reference	
Dry	7.67 (3.41–17.26)	<0.001	8.7 (4.05–18.70)	<0.001
***P. vivax***				
**Variable**	**OR (95% CI)**	***p* value**	**aOR (95% CI)**	***p* value**
Age group				
1–15 years	Reference		—	—
>15 years	0.89 (0.46–1.74)	0.743	—	—
Gender				
Female	Reference		Reference	
Male	2.78 (1.42–5.46)	<0.005	2.74 (1.34–5.62)	<0.01
Occupation				
Farmer	Reference		Reference	
Student and/or child	0.89 (0.44–1.80)	0.751	—	—
Other	1.32 (0.20–8.95)	0.775	—	—
Nights in the forest				
0–2	Reference		Reference	
3–7	9.03 (3.83–21.26)	<0.001	8.83 (3.78–20.60)	<0.001
8–14	36.04 (9.55–135.97)	<0.001	37.06 (9.64–142.53)	<0.001
>14	46.92 (4.61–477.20)	<0.005	44.93 (4.18–482.52)	<0.005
Season				
Monsoon	Reference		Reference	
Dry	0.88 (0.46–1.70)	0.706	—	—

OR unadjusted odds ratio, aOR adjusted odds ratio, CI confidence interval.

## Data Availability

The datasets generated and/or analyzed during the current study are not publicly available due to data protection from the Center of Malariology, Parasitology, and Entomology in Laos but can be available from the corresponding author with country approval.

## Supplementary Materials

The following supporting information can be downloaded at: https://www.mdpi.com/article/10.3390/ijerph21121624/s1, Table S1: Comparison of participants not missing the variable on nights spent in the forest vs. missing the variable on nights spent in the forest. Percentages are column percentages. Table S2: Sensitivity analyses for multivariable logistic regression.

## Abbreviations

ACTs	Artemisinin-based combination therapies
aOR	adjusted odds ratio
API	annual parasite index
CI	confidence interval
CMPE	Center for Malariology, Parasitology, and Entomology
DAG	directed acyclic graph
GMS	Greater Mekong Subregion
IRS	indoor residual spraying
Lao PDR	People’s Democratic Republic of Laos
LLIH	long-lasting insecticide-treated hammock
LLIN	long-lasting insecticide-treated net
OR	odds ratio
RCT	randomized controlled trial
RDT	rapid diagnostic test
*P. falciparum*	*Plasmodium falciparum*
*P. vivax*	*Plasmodium vivax*
WHO	World Health Organization
