# Supplementary material for: Forest-Going as a Risk Factor for Confirmed Malaria in Champasak Province, Lao PDR: A Case-Control Study
Source: Int J Environ Res Public Health. 2024 Dec 4;21(12):1624. doi: 10.3390/ijerph21121624 (PMC11675269; doi:10.3390/ijerph21121624)
Supplement: Supplementary file 1 [file ijerph-21-01624-s001.zip › ijerph-3224203-supplementary.pdf]

Table S1. Comparison of participants not missing the variable on nights spent in the forest vs missing the variable on nights spent in the forest. Percentages are column percentages.

	Not missing N = 2,933	Missing N = 3,306	Total N = 6,239
Malaria			
Control	2,689 (92%)	3,230 (98%)	5,919 (95%)
Case	244 (8%)	76 (2%)	320 (5%)
District			
Mounlapamoak	691 (24%)	1,120 (34%)	1,811 (29%)
Pathoumphone	1,474 (50%)	1,613 (49%)	3,087 (49%)
Sanasomboun	768 (26%)	573 (17%)	1,341 (21%)
Age group			
1-15 years	425 (14%)	636 (19%)	1,061 (17%)
>15 years	2,508 (86%)	2,670 (81%)	5,178 (83%)
Gender			
Male	1,984 (68%)	2,144 (65%)	4,128 (66%)
Female	949 (32%)	1,162 (35%)	2,111 (34%)
Occupation			
Farmer	2,294 (78%)	2,397 (73%)	4,691 (75%)
Student and/or child	511 (17%)	712 (22%)	1,223 (20%)
Other	128 (4%)	197 (6%)	325 (5%)
Season			
Dry	1,678 (57%)	1,819 (55%)	3,497 (56%)
Monsoon	1,255 (43%)	1,487 (45%)	2,742 (44%)

Table S2. Sensitivity analyses for multivariable logistic regression.

Variable	Sensitivity Analysis A: Overestimating forest sleeping[2]				Sensitivity Analysis B: Underestimating forest sleeping[3]			
	Number[4]				Number[4]			
	Malaria Negative N = 5,919	Malaria Positive N = 320	Adjusted OR (95% CI)	P value	Malaria Negative N = 5,919	Malaria Positive N = 320	Adjusted OR (95% CI)	P value
Age group								
1-15 years	1,014	47	—	—	1,014	47	—	—
>15 years	4,905	273	—	—	4,905	273	—	—
Gender								
Female	3,862	266	Reference	—	3,862	266	Reference	—
Male	2,057	54	2.63 (1.48-4.64)	0.001	2,057	54	2.52 (1.43-4.45)	0.001
Occupation								
Farmer	4,434	257	Reference	—	4,434	257	Reference	—
Student/child	1,175	48	0.84 (0.43-1.63)	0.60	1,175	48	1.00 (0.46-2.02)	0.91
Other	310	15	0.84 (0.34-2.04)	0.70	310	15	0.98 (0.41-2.35)	0.97
Nights in forest								
0-2	1,695	29	Reference	—	4,925	105	Reference	—
3-7	4,124	223	3.20 (1.83-5.60)	<0.001	894	147	7.91 (4.10-15.26)	<0.001
8-14	83	56	39.42 (12.73-122.11)	<0.001	83	56	32.18 (10.69-96.93)	<0.001
>14	17	12	42.80 (5.9-308.83)	<0.001	17	12	36.24 (5.26-249.78)	<0.001
Season								
Monsoon	2,637	105	Reference	—	2,637	105	Reference	—
Dry	3,282	215	1.68 (1.08-2.60)	0.02	3,282	215	1.75 (1.13-2.71)	0.01

OR: odds ratio; *CI:* confidence interval; [1] adjusting for gender, occupation, and season and with clustering at the health facility level; [2] sensitivity analysis to evaluate the impact on results if those missing data on forest sleeping had slept in the forest; [3] sensitivity analysis to evaluate the impact on results if those missing data on forest sleeping had not slept in the forest; [4] numbers include participants who were missing data on forest-sleeping.
